# Industrially benign super-compressible piezoresistive carbon foams with predefined wetting properties: from environmental to electrical applications

**DOI:** 10.1038/srep06933

**Published:** 2014-11-06

**Authors:** Tung Ngoc Pham, Ajaikumar Samikannu, Jarmo Kukkola, Anne-Riikka Rautio, Olli Pitkänen, Aron Dombovari, Gabriela Simone Lorite, Teemu Sipola, Geza Toth, Melinda Mohl, Jyri-Pekka Mikkola, Krisztian Kordas

**Affiliations:** 1 Technical Chemistry, Department of Chemistry, Chemical-Biological Centre, Umeå University, SE-90187 Umeå, Sweden; 2 Microelectronics and Materials Physics Laboratories, Department of Electrical Engineering, University of Oulu, P.O. Box 4500, FI-90014 University of Oulu, Finland; 3 Industrial Chemistry & Reaction Engineering, Department of Chemical Engineering, Process Chemistry Centre, Åbo Akademi University, FI-20500, Åbo-Turku, Finland

## Abstract

In the present work electrically conductive, flexible, lightweight carbon sponge materials derived from open-pore structure melamine foams are studied and explored. Hydrophobic and hydrophilic surface properties - depending on the chosen treatment conditions - allow the separation and storage of liquid chemical compounds. Activation of the carbonaceous structures substantially increases the specific surface area from ~4 m^2^g^−1^ to ~345 m^2^g^−1^, while retaining the original three-dimensional, open-pore structure suitable for hosting, for example, Ni catalyst nanoparticles. In turn the structure is rendered suitable for hydrogenating acetone to 2-propanol and methyl isobutyl ketone as well for growing hierarchical carbon nanotube structures used as electric double-layer capacitor electrodes with specific capacitance of ~40 F/g. Mechanical stress-strain analysis indicates the materials are super-compressible (>70% volume reduction) and viscoelastic with excellent damping behavior (loss of 0.69 ± 0.07), while piezoresistive measurements show very high gauge factors (from ~20 to 50) over a large range of deformations. The cost-effective, robust and scalable synthesis - in conjunction with their fascinating multifunctional utility - makes the demonstrated carbon foams remarkable competitors with other three-dimensional carbon materials typically based on pyrolyzed biopolymers or on covalently bonded graphene and carbon nanotube frameworks.

Porous carbon materials, prepared from biomaterials, have been utilized over hundreds of years, mainly in medicine, environmental work and food processing in order to mitigate or completely eliminate pollutants of chemical and biological origin. The commonly exploited properties in these applications are the high specific surface area and associated surface adsorption and open pore structure enabling good permeation of the pores with gaseous and liquid media^1^. Polyurethane^2^ and phenolic^3^ foams, as well as aerogels based on resorcinol-formaldehyde^4^,^5^, have been in the focus of contemporary research owing to the tunable physicochemical properties of the derived carbon foams enabling low density, good mechanical strength and high surface area of the mentioned materials. Recently, 3-dimensional mechanically flexible structures - a novel family of porous carbon materials - synthesized from carbon nanotubes^6^, graphene^7^, graphene oxide^8^ and their composites^9^,^10^ have attracted considerable attention for their advanced application as soft low-noise electrical contacts^11^, gas/liquid storage^12^, supercapacitor^13^ and solar cell electrodes^14^, particulate filters as well as catalyst support membranes^15^.

Although each of the above-mentioned 3-dimensional porous carbons possess several fascinating properties, the pyrolyzed polymers are lacking good electrical conductivity, mechanical integrity and flexibility, while carbon nanotube and graphene based foams are difficult to synthesize in bulk quantities, thereby limiting their practical use and commercialization.

Very recently, a new class of flexible carbon foams was reported to have been prepared by the direct carbonization of melamine foams^16^, thus opening up new avenues for truly multifunctional porous carbon materials produced on a fully-enabled large scale. Based on their excellent properties - such as light weight, high porosity, hydrophobicity and high adsorption capacity towards polar organic liquids - such foams were found to have many promising applications as flexible electrodes or as sorbent material in water purification. Moreover, these novel spongy carbons were successfully applied as a catalyst for wet air oxidation of aniline^17^ or as a carrier for N-doped Ketjenblack® used in oxygen reduction reaction^18^. Obviously, this flexible open pore structure carbon material, derived from melamine foam, possesses unique properties; consequently, it is envisioned as playing an important role in various kinds of industrially relevant applications, and thus there is an instant need to investigate further such a promising material in order to explore and completely exploit its advantageous properties.

In this paper, we show that after the pyrolysis and activation process, the carbon foam retains the open pore network of the precursor foam and remains mechanically flexible, and, furthermore, with a proper selection of suitable synthesis conditions, foams possessing a different hydrophobic/hydrophilic nature may be obtained. X-ray photoelectron analysis reveals the formation of polar surface groups in the samples following pyrolysis at high temperatures, which clearly explains the hydrophilic behavior. When activated, the specific surface area can be increased by almost two orders of magnitude while still retaining its hydrophilic nature, thus offering an excellent porous platform material to synthesize and support catalyst nanoparticles in the porous framework. Ni nanoparticles deposited in the pores by means of wet impregnation are demonstrated as efficient catalyst materials to convert, for example, acetone to 2-propanol. Furthermore, we also demonstrate that carbon foams can serve as a scaffold for hierarchical carbon nanostructures. Using the Ni decorated foams we grow carbon nanotubes in the porous structures and demonstrate novel types of electrochemical electrodes, thus extending the utility of these materials. Furthermore, we show that the electrical conductivity of the pyrolyzed foams can be significantly tuned by mechanical deformation indicating that the foams are highly piezoresistive and suitable for mechanical strain sensing. These combined properties make such carbon foams truly multipurpose and allow diverse applications including, but not limited to, adsorbers, catalyst supports, flexible electrodes, strain gauges as well as mechanical vibration damping components.

## Results and Discussion

### Microstructure, surface chemistry and wetting behaviour

**Figure 1:**
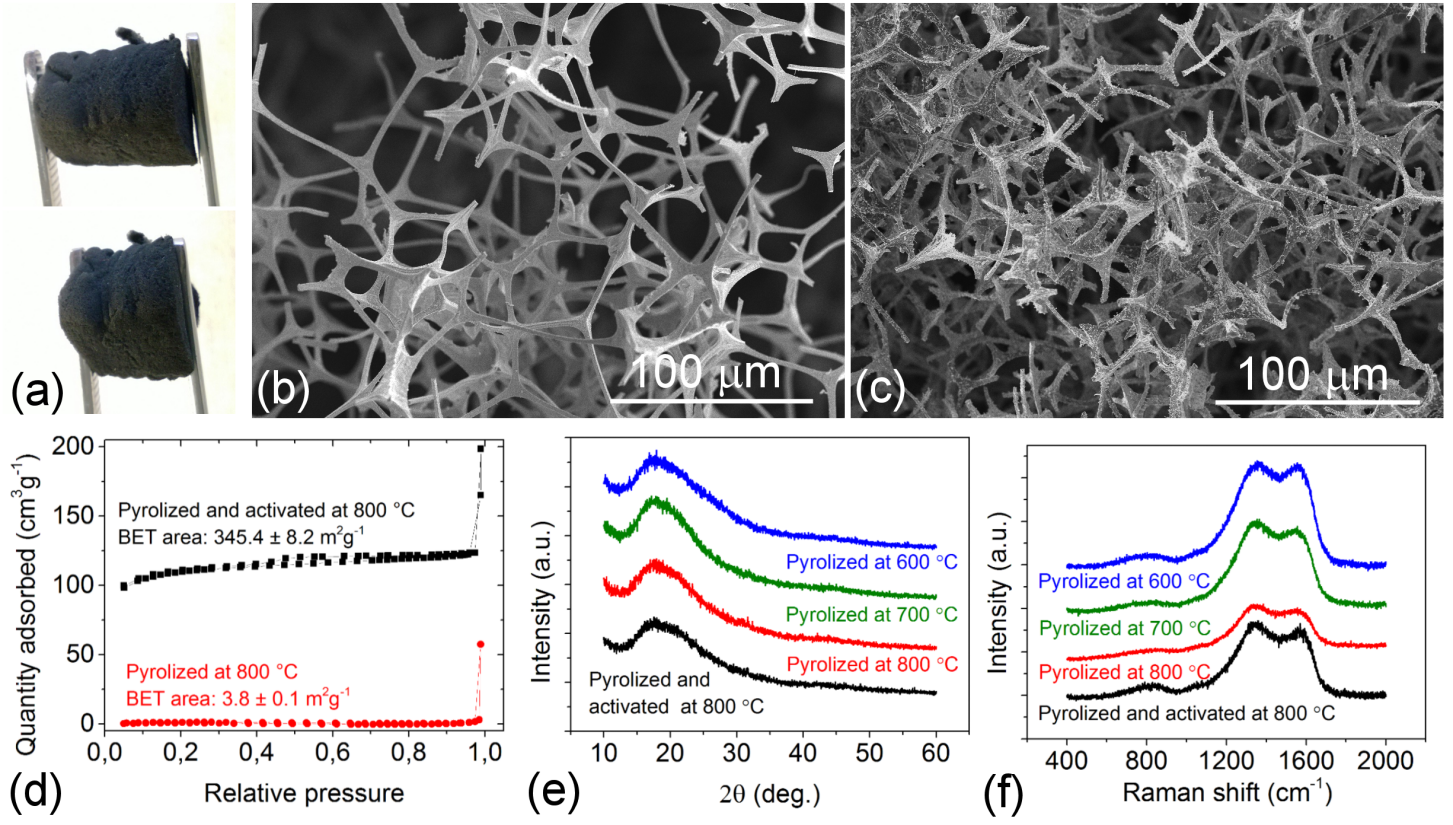
(a) Digital camera image of a piece of pyrolyzed foam as pressed by tweezers. Panels (b) and (c) show scanning electron micrographs of foams pyrolyzed at 800°C in N_2_ and subsequently activated in 2% CO_2_ in N_2_. (d) BET isotherms of the corresponding samples. (e) X-ray diffraction patterns of foams synthesized under different conditions. Note: The diffuse X-ray scattering at 2θ ~18° is due to the glass substrate and Scotch tape used for mounting the samples in the fixture. For clarity, the diffraction patterns are shifted vertically. (f) Raman spectra of the foams synthesized under different conditions. Note: the spectra are normalized to the same background and shifted vertically for comparison and clarity, respectively.

Due to the nature of the pyrolysis process, the original melamine foam lost ~70–80% of its weight and shrunk by 30–40% compared to its original size, resulting in a density of ~8 mg·cm^−3^, albeit rather similar to the value of the starting material (~9 mg·cm^−3^). Unlike other 3-dimensional porous carbon media – typically obtained by pyrolyzing biomass – the melamine-based sponges remain flexible while preserving their integrity following pyrolysis (Fig. 1a). According to scanning electron microscopy analyses (Fig. 1b and c), the skeletal structures of the pyrolyzed foams are very similar to that of the original polymer sponge. After pyrolysis, even at the highest of temperatures, the surface area of the samples remained quite low (~4 m^2^g^−1^) (Fig. 1d) in keeping with the result of Sousa et.al (0.5 m^2^g^−1^)^17^ but contradicting the values reported by Chen et.al (268 m^2^g^−1^)^16^. This difference may be explained by the different types of precursor foams (e.g. Basotect® this work and ref. 17 versus SINOYQX in ref. 16).

On the other hand, by activating the carbon foam in CO_2_, we obtain a novel flexible hydrophilic carbon foam which is rather different from the hydrophobic material reported earlier^16^. The hydrophilic nature of the monolith-type carbon sponge is because of polar surface functional groups (Figure S2) forming during the activation process, which in turn is efficient in the immobilization of metal ions.

Activation in CO_2_ further structures the skeletal material by introducing voids and small fibrils growing in the carbonaceous scaffold, thus increasing the relative surface area of the material by two orders of magnitude, from ~4 m^2^g^−1^ to ~345 m^2^g^−1^, as revealed by N_2_ surface adsorption measurements (Fig. 1d).

In the course of pyrolysis, all of the sulfur and most of the oxygen content are removed, leading to an increasing relative atomic percentage of carbon and nitrogen on the surface (Fig. S1). At the same time, the transformation of less thermally stable amines (399.5 eV) into other functionality forms of nitrogen which are more thermally stable, such as pyridine-N (398.1–398.4 eV) and quaternary-N (Table 1), was observed^19^,^20^,^21^. According to Tiva et.al^20^, based on the differences of the binding energy, quaternary-N can be divided into center-N (401 eV) and valley-N (402.3–402.6 eV). In our sample, while the peak of valley-N is quite easy to isolate, the peak of center-N is not so clear and can be mixed with the signal from pyrrolic-N (400–400.3 eV^19^,^20^), especially for samples at low pyrolyzed temperatures (Table 1).

As shown in Fig. S1, sodium is present on the surface of all samples, especially for foams pyrolyzed above 700°C. The surface of foams pyrolyzed above 700°C is enriched with Na due to the migration of ionic impurities to the surface, which significantly affects the wetting properties of the carbon foam. For instance, a sample pyrolyzed at 800°C displays ~16 at.% Na on its surface, and illustrates well the highly hydrophilic nature of such foams (Video S1). Once the sample is exposed to air, the sodium ions can react with water and CO_2_ in air to form e.g. Na_2_CO_3_ and/or NaHCO_3_ (peaks from 1071.1–1071.5 eV) (Table S1) making it easy to wet the surface with water (Video S1). However, after soaking and washing the pyrolyzed (800°C) samples several times with water, a major part of the sodium and oxygen can be removed from the surface (Fig. S1), changing it from hydrophilic to hydrophobic. It is important to note, however, that for the activated sample, due to the formation of polar groups on the surface (Fig. S2), the hydrophilic nature remains unchanged even after washing in water.

**Figure 2:**
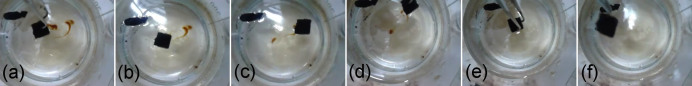
(a) to (f) Sequence of video frames showing the removal of crude oil from water by the means of selective adsorption using carbon foam pyrolyzed at 600°C.

The hydrophobic samples are capable of taking up large amounts of non-polar solvents up to ~100-times the weight of the dry foam (Table 2). In Fig. 2 and Video S2, removal of crude oil from the surface of water is demonstrated by applying pieces of hydrophobic (and also oleophilic) carbon foams synthesized at 600°C. The hydrophobic foam pieces float on the top of the surface and collect oil patches from it when brought into contact with them. The used carbon foams may be recycled by squeezing oil/solvent out of them. Burning the organic liquids is another approach to refurbishing the foams. However, in such cases, the flexibility of the structure is lost.

### Carbon foams as versatile catalyst support

In solid heterogeneous catalysis one of the most important criteria for a good support material is a high specific surface area that facilitates the accessibility of the metal catalysts. Accordingly, activated samples with a specific surface area of ~345 m^2^g^−1^ were considered in demonstrating this functionality of the carbon sponge. The activation process not only brings a high specific surface area to the material, but also results in polar surface functional groups (Figure S2) that are efficient in the immobilization of metal ions. The sponge's ability to trap metal ions from aqueous solutions of Ni(NO_3_)_2_ was found to be ~10 wt. % (by ICP-OES) of the adsorbent weight.

**Figure 3:**
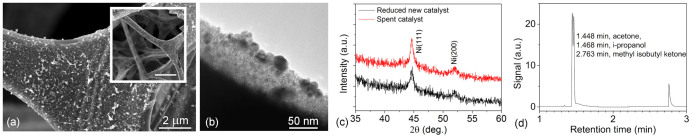
Microstructure of the Ni decorated carbon sponge shown in (a) scanning and (b) transmission electron micrographs (scale bar in inset is 10 μm). (c) X-ray diffraction patterns of fresh and spent catalyst. (d) Chromatogram of the acetone hydrogenation reaction products obtained at 250°C on activated and Ni decorated carbon foam.

Based on the broadening of the Ni(111) and Ni(200) peaks (at 44.6° and 52.0°, respectively) and reflections in the X-ray diffraction (XRD) patterns (Fig. 3), the average crystal size after reduction was calculated to be 10.8 ± 3.2 nm, which is in good agreement with the average catalyst size assessed by transmission electron microscopy (9.2 ± 5.0 nm). After the model reaction (namely, hydrogenation of acetone to 2-propanol), the average crystal size of nickel did not show any significant change (11.6 ± 3.9 nm according to XRD), indicating that the catalyst is well immobilized and not prone to either diffusion or sintering. Interestingly, upon the metal decoration process, the tiny fibrils that formed during the activation of the carbon sponge become detached from the structure and at some surface locations peeling of the carbonaceous scaffold is also observed.

**Figure 4:**
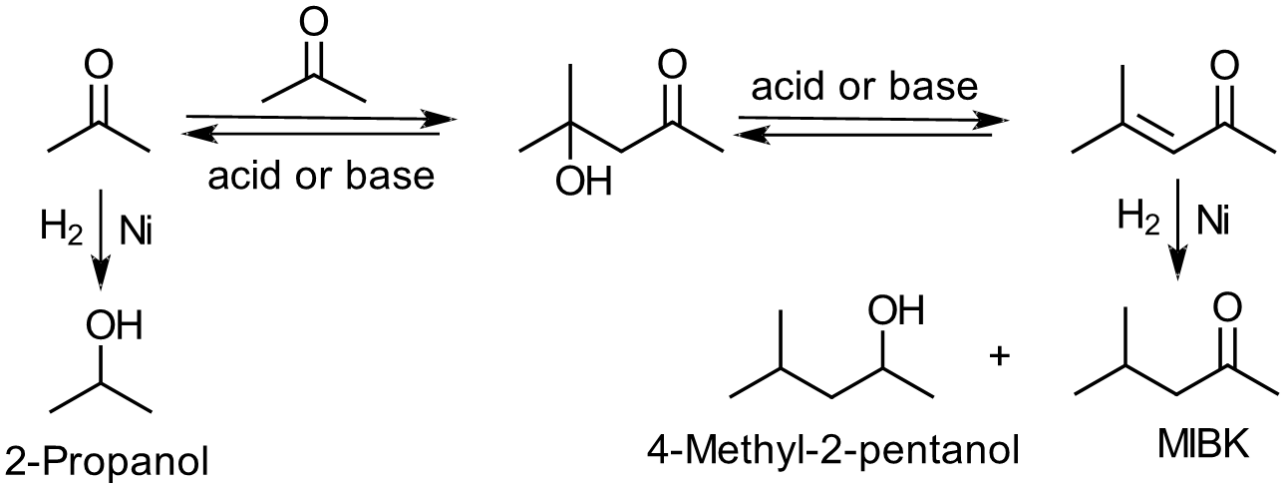
Possible reaction paths during the catalytic hydrogenation of acetone^22^,^23^,^24^,^25^.

Although the distribution of Ni catalyst particles on the microscopic wires of the carbon scaffold is not entirely homogeneous (Fig. 3), the monolith-type catalyst sponge performs well in catalytic hydrogenation of acetone vapor at 150°C. Even though parameters such as reaction temperature, reactant flow rate and catalyst amount have not been optimized, conversion of 86% and selectivity of ~99% for 2-propanol were reached and traces of methyl isobutyl ketone (MIBK) and 4-methyl-2-pentanol were detected (determined by GC and GC-MS) (Fig. 3(d) and Table 3). At higher reaction temperatures (250°C), as shown in Fig. 3(d), however, the formation of MIBK is also favored (selectivity of 11%), indicating that the carbon-supported Ni catalyst is also active in promoting aldol condensation^22^,^23^,^24^,^25^ (Figure 4), which is usually performed over catalyst metals supported on zeolites^22^, talcite^23^ and alumina^24^. Since the reaction requires the presence of metal catalysts as well as acid or basic surface sites on the support, the basic pyridinic (peak at 398.1 eV) and quaternary nitrogen (peaks at 401.0 and 402.5 eV) (Table S1) functionalities on the surface of the carbon foam can act as catalytic sites promoting the aldol reaction similar to N-doped carbon nanotubes used in Knoevenagel condensation^26^. Accordingly, the carbon foam can be used not only as a support material but also as a co-catalyst for chemical reactions.

Compared to other common powder-type catalyst supports (such as zeolites, silica and activated carbon), the superiority of our carbon foam is based on its flexible monolith-type nature. For these powder-type catalyst supports, a tube reactor with special design and additional supporting materials (typically glass beads or quartz wool) are needed to keep the active materials in place and to avoid densification. Since the flow inside the catalyst bed is highly influenced by the way of packing, much room for error may be present. However, with the monolith-type catalyst foams, due to their flexible nature, the catalyst bed can be directly mounted in flow-through reactors without the need of any additional means of immobilization to retain the catalytic material. This inherently simplifies the process, improves repeatability, and reduces costs associated with packing and recovering the catalytic materials.

### Electrical transport and electrochemical energy storage

**Figure 5:**
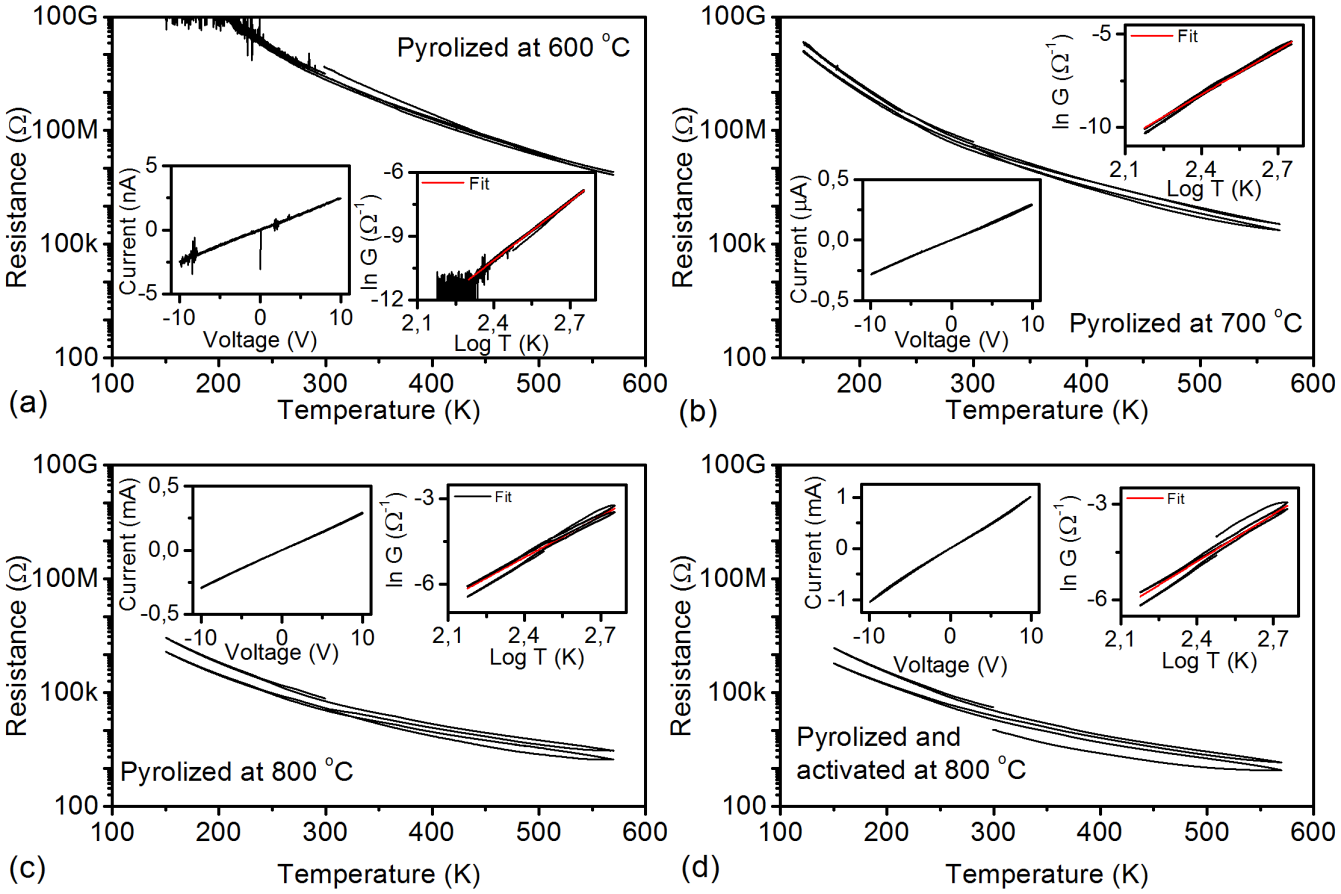
Electrical properties of the carbon foams. Resistance-temperature sweeps, current-voltage sweeps (left inset), and multi-phonon assisted hopping model fittings of resistance-temperature sweeps for samples pyrolyzed at (a) 600°C, (b) 700°C, (c) 800°C, and (d) pyrolyzed as well as activated at 800°C. Insets in the right hand side of each graph display the linearized form of experimental conductivity vs. temperature data along with the corresponding MPH fitting curves.

The temperature applied for pyrolysis plays a major role in the electrical conductivity of the samples as indicated by the ~5 orders of magnitude decrease of the measured resistance for the samples pyrolyzed at 800°C, as compared to those processed at only 600°C (Fig. 5). For each carbon sponge, a negative temperature coefficient of resistance is observed, thus the materials cannot be considered as porous metallic conductors despite their linear current-voltage behavior (see left-side insets in panels of Fig. 5). The temperature dependence of resistance seems to follow the model for variable-range hopping (VRH) in 3-dimensions^27^,^28^,^29^,^30^,^31^,^32^, however, the calculated localization length and density of electronic states give unrealistic values (see Supplementary Information). Instead, multiphonon hopping (MPH) is assumed to be governing the charge transfer mechanism^33^. In MPH, the resistivity is proportional to the power function of temperature as *σ* ~ (*T*/*T*_0_)*^p^*, where *p* denotes the numbers of coupled phonons involved in the hopping process. The resistance-temperature dependency of the measured data obeys the above relationship well (see right-side insets in panels of Fig. 5) having reasonable values for *p* from 9.2 to 4.8 for samples pyrolyzed at 600°C and 800°C, respectively.

MPH is a rather plausible explanation for the mechanism of electrical conduction in our samples since in the course of the pyrolysis process, the porous polymer sponge undergoes carbonization, which results in the formation of nanoscopic conductive patches with delocalized electrons as well as localized electron states - in the forbidden band of the polymer – that allow hopping of electrons by the means of tunneling processes between adjacent states of random spatial and energetic spacing. Surprisingly, graphitization does not take place in the studied temperature range according to X-ray diffraction (Fig. 1e) and Raman spectroscopy (Fig. 1f). The diffraction patterns show completely diffuse scattering originating from the sample holder. The lack of characteristic (002) planes of graphite suggests very poor ordering (if any) of the sp^2^ carbons detected by Raman spectroscopy.

**Figure 6:**
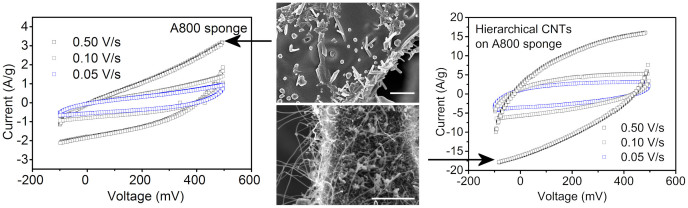
Electric double-layer capacitance behavior of the carbon foams in KOH electrolyte. Current-voltage behaviour of supercapacitors using carbon sponge electrodes pyrolyzed and activated at 800°C (left panel) and hierarchical carbon sponge-nanotube structures (right panel). Field-electron microscopy images in the middle panels show the corresponding carbon structures (scale bars are 2 μm).

To further explore the potential of the carbon foams, we have tested the porous gas permeable samples as templates to grow carbon nanotubes by low-pressure catalytic chemical vapour deposition from acetylene using Ni catalyst anchored in the pore walls by wet impregnation. After a period of 30 min synthesis carbon nanotube films with a thickness of ~2 μm grow on the skeletal structure of the foam forming hierarchical carbon architecture (Fig. 6).

Supercapacitor structures were made by using the most conductive sponge, i.e. pyrolyzed and activated at 800°C, as well as using the hierarchical carbon sponge-nanotube samples (Fig. 6). For the carbon sponge electrodes, the measured cyclic voltammetry curves show hysteresis, however the charging current is not saturating – as would be expected from an ideal capacitor - due to the large series resistance of the component caused by the limited conductivity of the electrodes. The curves are somewhat asymmetric, due to the non-equal masses of the electrodes. The overall specific capacitance of the electrodes is in the range of ~10 F/g, which is reasonable considering the specific surface area (>100 m^2^/g) of the materials^34^,^35^. On the other hand, the electrodes made of the hierarchical carbon sponge-nanotube materials provide more rectangular C–V curves and considerably higher specific capacitances (~40 F/g) than the reference sponge materials, which is a consequence of the more easy-to-access surface for the ions of the electrolyte as well as the improved overall conductivity of the 3D structure.

### Super-compressible piezoresistive structures

**Figure 7:**
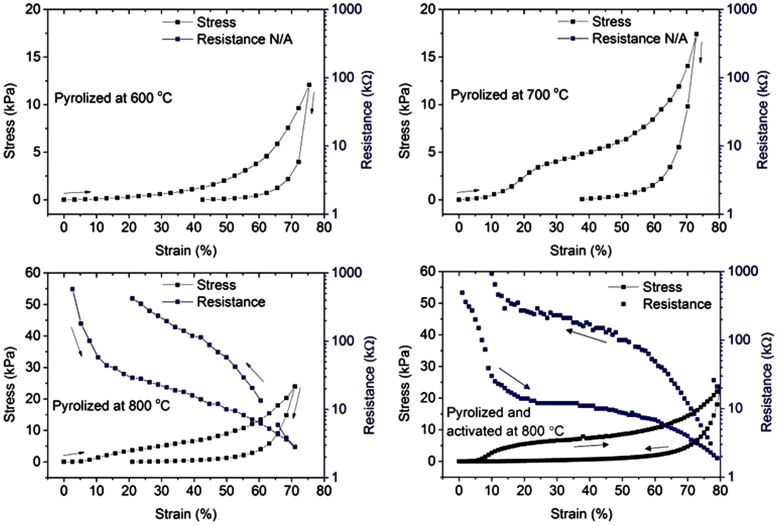
Stress-strain and resistance-strain (piezoresistive) behaviour of carbon foams pyrolyzed at (a) 600°C, (b) 700°C, (c) 800°C and (d) pyrolyzed and activated at 800°C.

Each sample is highly flexible and compressible with reversible deformation (up to ~75% strain). Stress-strain measurements (Fig. 7) show that the foams become stiffer when pyrolyzed at higher temperatures. The Young's moduli at low strain regime (i.e. between 5% and 20% compressive deformation) are Y_P,600_ = 1.5 ± 0.1 kPa, Y_P,700_ = 9.6 ± 1.4 kPa, Y_P,800_ = 24.7 ± 1.5 kPa and Y_P+A,800_ = 46.3 ± 2.0 kPa, respectively. The stress-strain curves exhibit a plateau between 20% and 40% strain and then show a gradually increasing steep slope above compression of ~40% - similar to those of other compressible foams and sponge-like materials^36^,^37^,^38^,^39^ – caused by the densification of the structures^40^,^41^,^42^. Despite the very low strain rates applied (<0.1%/s) the stress-strain curves have considerable hysteresis indicating processes associated with energy losses (0.69 ± 0.07), e.g. friction and structural rearrangement, that are typical when deforming foams, suggesting applications for noise isolation, mechanical damping and shock absorption. (It is worth mentioning that upon repeated deformation tests, the foams undergo softening as displayed in Fig. S4).

The resistance-strain plots (Fig. 7) exhibit a nearly exponential slope with almost three orders of magnitude drop of resistance upon 70% compressive strain. Strain-induced change of carrier mobility similar to that in semiconductors is unlikely to be the reason for such a considerable piezoresistive effect. The significant increase of conductance is probably due to an enhanced percolation of conductive volumes observed for nanostructured carbons earlier^43^. Since the resistance-strain curves are highly nonlinear, the corresponding gauge factors (GF) depend on the strain. The highest values measured for GF are between ~20 and 50 (under pre-stressed conditions) being comparable with other carbon-based strain gauges, e.g. suspended CNT networks (GF ~ 75 ± 5)^44^, epoxy-graphite composites (GF ~ 10–45)^45^ and CVD grown diamond films (GF up to ~30)^46^.

## Conclusions

In conclusion, we have shown truly multifunctional flexible carbon foams derived from self-similar open pore structure melamine foams in this work. Depending on the sample treatment conditions, with or without subsequent activation, either hydrophobic or novel hydrophilic foams could be synthesized making the materials suitable for absorbing/repelling solvents of various polarities. The novel hydrophilic carbon foams proved to be efficient monolith-type catalyst materials as demonstrated by gas-phase hydrogenation of acetone to 2-propanol and methyl isobutyl ketone as well as by growing hierarchical carbon nanotube structures using the 3-dimensional carbon scaffolds as templates. Mechanical stress-strain measurements revealed very high compressibility with viscoelastic behavior that may be exploited in vibration damping and shock absorption applications. Furthermore, the very high piezoresistive gauge factors (between 20 and 50) assessed by resistance-strain measurements suggest use of these materials in robust but ultra-sensitive strain gauges. The results of the current study indicate that carbonization and subsequent chemical modifications of the porous foams can lead to a range of different novel multifunctional materials with engineered physical and chemical properties that, in turn, have great potential to be exploited in environmental applications, catalytic converters, growth templates for hierarchical nanostructures, porous high surface area electrode materials, mechanical vibration damping and sensitive displacement/strain sensing.

## Methods

### Carbon foam synthesis

Melamine-based polymer foam (BASF, Basotect® W, used as received) was pyrolyzed at temperatures between 600 and 800°C (1 hour with the ramping rate of 1°C/min) in a quartz reactor under N_2_ flow (50 mL/min). Consequently, selected samples pyrolyzed at 800°C were also activated at 800°C with CO_2_ (1 mL/min, 2% by volume in N_2_) in 2 hours. After the heat treatment process, the system was allowed to cool down to room temperature in inert atmosphere (N_2_).

### Materials characterization

X-ray photoelectron spectroscopy (XPS, Axis Ultra DLD spectrometer with a monochromatized Al Kα X-ray source with charge neutralization) was used to analyze the chemical composition of the samples. The microsctructure of the specimens were studied by field emission scanning electron microscopy (FESEM, Carl Zeiss SMT MERLIN at 3 kV) and by transmission electron microscopy (EFTEM, LEO 912 OMEGA at 120 kV using LaB_6_ filament). Crystal phase assessment of the carbon foams was performed with X-ray diffraction (XRD, Bruker D8 Discover, Cu Kα radiation source) and micro-Raman spectroscopy (Horiba Jobin-Yvon LabRAM HR800, Ar^+^ laser source at λ = 488 nm). BET surface area measurements were carried out by N_2_ adsorption analysis (Tristar 3000 apparatus, Micrometrics Instrument Corp.).

### Oil/Solvent absorption capacity analysis

To identify the liquid absorption capacity, carbon foams pyrolyzed at 600°C were immersed into silicone oil, benzene, turpentine oil, crude oil and isohexane for ~20 seconds. The adsorption capacity, i.e. the change of mass in reference to the original mass of the dry sponge, was calculated from gravimetric measurements. The final results were averaged out from three tests.

### Electrical measurements

Four different kinds of carbon sponge samples (pyrolyzed at 600, 700 and 800°C, respectively, as well as pyrolyzed and activated at 800°C) were analyzed by the means of current-voltage, resistance-temperature, cyclic voltammetry, stress-strain and piezo-resistive measurements.

The samples used in the electrical and mechanical measurements were cut to a size of ~5 × 5 × 3 mm^3^ with a surgical blade. In the course of the current-voltage and resistance-temperature studies, the samples were placed over Au electrodes (screen printed on an alumina substrate, electrode spacing of ~200 μm and length of ~3 mm). To ensure an intimate contact with the electrodes, the sponges were slightly pressed against the substrate from the top. The gold electrodes were probed in a Linkam THMS600 stage and connected to a Keithley 2612 SourceMeter. All the electrical measurements were performed in air. Current-voltage sweeps were performed from −10 V to 10 V with 0.2 V/s scanning speed at 30°C. In addition, resistance-temperature sweeps were performed with constant bias of 10 V from 150 K to 570 K with a heating/cooling rate of 10 degrees per minute.

Cyclic voltammetry measurements were carried out for the samples carbonized at 800°C. Other samples synthesized at lower temperatures were omitted because of their low conductance observed in current-voltage measurements. Two similar carbon foams were stacked between two stainless steel plates (Inconel® 600, thickness of 75 μm and composition of Ni 72%, Cr 16% and Fe 8% from Goodfellow) and separated with a filter paper (Whatman 1, Cat. No 1001-047). Before stacking, platinum of about 25 nm thickness was sputtered on one facet of each carbon sponge block to ensure a uniform electrical contact with the Inconel® plates. The stack was impregnated with an electrolyte of 4:1 vol. mixture of aqueous KOH (6 mol/L) and isopropyl alcohol. The stack was connected to a Princeton Applied Research VersaSTAT3 potentiostat using crocodile jaws clamping the Inconel® sheets. Current-voltage curves were recorded from −100 mV to 500 mV by using 0.05, 0.1, 0.5 and 1 V/s scanning speeds.

### Mechanical and piezoresistive measurements

The mechanical stress-strain and piezoresistive properties were assessed by placing the samples between copper surfaces on a bench consisting of a weighting scale and a computer controlled vertical translation stage. The pressing force was calculated from the measured weight upon compression adjusted by the vertical displacement of the upper copper electrode plate. The copper plates were connected to a Keithley 2612 SourceMeter providing a constant 10 μA current through the samples while measuring the voltage drop.

### Catalyst synthesis and activity testing

For catalytic activity experiments, Ni decorated carbon foams were synthesized. In a typical process, activated carbon sponge was immersed in a solution of Ni(NO_3_)_2_ for 24 hours (with ~400 ppm initial concentration of Ni^2+^). To determine the amount of Ni^2+^ adsorbed on the carbon foam surface, the solution (before and after adsorption) was diluted 10 times in 3% HNO_3_ and measured by using ICP-OES instrument (PerkinElmer OPTIMA 2000 DV). The as-obtained impregnated carbon foam was dried in air at 100°C for 24 h and then reduced in H_2_ flow at 400°C for 2 hours.

As a model reaction, the reduction reaction of acetone to isopropanol was carried out in hydrogen atmosphere in a glass tube reactor. Around 0.2 g of the sample (Ni decorated carbon foam) was mounted in the center of the glass tube reactor (inside diameter 1.3 cm and length 35 cm). Acetone was pumped into the reactor with a feeding rate of ~5 μL/min and mixed with hydrogen (~2 mL/min) at a specified temperature (150 and 250°C). The vapor phase product was condensed and the liquid was collected for 2 hours of reaction to perform gas chromatography (GC) analysis (Agilent Technologies, 7820A equipped with a HP-PONA capillary column and FID detector) and also GC (7820A equipped with a HP-5 capillary column) coupled with mass spectroscopy (5975 series MSD).

The Ni decorated carbon foams were also tested for growing carbon nanotubes from acetylene precursor by using catalytic chemical vapor deposition (30 min growth time, at a temperature of 670°C, pressure of 500 mbar with gas flows of 700 sccm H_2_, 500 sccm N_2_ and 5 sccm C_2_H_2_) in a cold wall reactor (Aixtron Black Magic).

## Supplementary Material

Supplementary Information Supplementary information

Supplementary Information Video S2 Crude oil separation from water

Supplementary Information Video S1 Hydrophilic vs. hydrophobic carbon foam

## Figures and Tables

**Table 1:** N1s X-ray photoelectron peaks and their assignment for the pristine polymer foam (BST), carbon foams after pyrolysis at various temperatures (P600–P800), activation (A800) and subsequent soaking in water (P800* and A800*)

BST		P600		P700		P800		P800*		A800		A800*		
BE, eV	AC, at.%	BE, eV	AC, at.%	BE, eV	AC, at.%	BE, eV	AC, at.%	BE, eV	AC, at.%	BE, eV	AC, at.%	BE, eV	AC, at.%	Assignment
						396.7	0.5			395.9	0.5			Dielectric phase
398.3	6.8	398.1	13.5	398.4	11.5	398.2	3.7	398.1	8.2	398.2	4.9	398.1	6.5	Pyridinic-N
399.5	11.4	399.2	2.7	399.6	2.1	399.5	1.9	399.7	4.1	400.2	1.3	399.9	6.3	Amine-N (may be mixed with pyrrolic-N
		400.6	7.3	400.9	6.1	400.8	2.5	400.8	3.4	401.4	0.6	401.0	1.8	Quaternary-N (center-N) (may be mixed with pyrrolic-N)
		402.6	1.6	402.8	1.7	402.6	1.1	402.3	1.3			402.5	1.2	Quaternary N (valley-N)
404.8	0.8	404.9	0.9	405.1	0.8			404.4	0.6			405.0	0.7	π- π* excitation

**Table 2:** Uptake of nonpolar organic liquids by carbon foams synthesized at 600°C. The quantity Δm/m_0_ denotes the mass gain normalized to the starting weight of the dry carbon sponge

Liquid (and its density)	Δm/m_0_
Silicone oil (1.402 g/cm^3^)	106 ± 1
Benzene (0.879 g/cm^3^)	101 ± 8
Turpentine oil (0.856–0.867 g/cm^3^)	95 ± 3
Crude oil (0.847–0.862 g/cm^3^)	79 ± 2
Iso-hexane (0.653 g/cm^3^)	77 ± 6

**Table 3:** Temperature effect on the conversion and product selectivity for acetone hydrogenation using Ni decorated carbon foams determined from the relative peak area of gas chromatograms. The turnover frequency for acetone to 2-propanol conversion at 150°C is ~0.1 molecule/site/s

		Selectivity		
Temperature	Acetone conversion	2-propanol	MIBK	4-methyl-2-pentanol
150°C	86%	99%	0.4%	0.4%
250°C	44%	86%	11%	1%
