# Supplementary material for: Industrially benign super-compressible piezoresistive carbon foams with predefined wetting properties: from environmental to electrical applications
Source: Sci Rep. 2014 Nov 6;4:6933. doi: 10.1038/srep06933 (PMC5381471; doi:10.1038/srep06933)
Supplement: Supplementary Information [file srep06933-s1.pdf]

Supporting Information

Industrially benign super-compressible piezoresistive carbon foams with predefined wetting properties: from environmental to electrical applications

Tung Ngoc Pham, Ajaikumar Samikannu, Jarmo Kukkola, Anne-Riikka Rautio, Olli Pitkänen, Aron Dombovari, Gabriela Simone Lorite, Teemu Sipola, Geza Toth, Melinda Mohl, Jyri-Pekka Mikkola, and Krisztian Kordas

Evaluation of variable range hopping model for the 3D carbon sponge samples: Although the temperature dependence of resistance can be fitted rather well with model for variable-range hopping (VRH) in 3-dimensions, we need to calculate the values for localization length and density of electronic states to validate the model. The temperature dependence of resistance in 3D VRH 1 is $\sigma = \sigma_0 e^{-(T_0/T)^{1/4}}$, where $T_0 = 18(k\xi^3 N_E)^{-1}$, k is the Boltzmann constant, ξ is the radius of the localized electron wave function (or the localized states) and N_E is the density of states around Fermi level, $\sigma_0 = \frac{3e^2 \nu_{ph}}{(8\pi)^{1/2}}\left[\frac{\xi N_E}{T}\right]^{1/2}$, e is the elementary charge, and ν_{ph} is the phonon frequency associated with hopping process (~10^{13}Hz). Each $\ln(GT^{1/2})\ vs\ T^{-1/4}$ plot of the measurement data can be fitted well with a linear function (not shown here), from which the extracted values of T_0 fitting parameters are between $7.8\cdot10^8$ K and $6.4\cdot10^7$ K for the samples pyrolyzed at 600°C and 800°C, respectively. The results match well with values reported earlier in the literature e.g. for amorphous carbon (7×10^7 K) 1-4 or for silicon suboxides (SiO_x, where x=0.01-1.82 ($6.3\cdot10^7$ - $6.0\cdot10^9$ K) 5. In addition, based on the fittings and the conductivity of the carbon sponges (evaluated by a parallel plate setup with rectangular sponges), localization length ξ and density of states $N(E_F)$ were evaluated to be from 8.6×10^{-29} m to 7.5×10^{-25} m and from 2.5×10^{99} to 4.8×10^{88} $m^{-3}J^{-1}$ to samples pyrolyzed at 600°C and 800°C, respectively. These values are unphysical (despite the excellent fit in the $\ln(GT^{1/2})\ vs\ T^{-1/4}$ plots) thus VRH is unlikely to explain the conductivity in the films.

1. Prins, F.J. Onset of hopping conduction in carbon-ion-implanted diamond, *Phys. Rev. B* **31**, 2472-2478 (1985).
2. Skákalová, V., Kaiser, A.B., Woo, Y.S., Roth, S. Electronic transport in carbon nanotubes: From individual nanotubes to thin and thick networks, *Phys. Rev. B* **74**, 085403 (2006).
3. Vishwakarma, P.N., Subramanyam, S.V. Hopping conduction in boron doped amorphous carbon films, *J. Appl. Phys.* **100**, 113702 (2006).
4. Hauser, J.J. Hopping conductivity in amorphous carbon films, *Solid State Commun.* **17**, 1577-1580 (1975).
5. van Hapert, J.J. Hopping Conduction and Chemical Structure: a study on Silicon Suboxides, 2002, University of Utrecht.

XPS analysis of carbon foam samples used in this work:

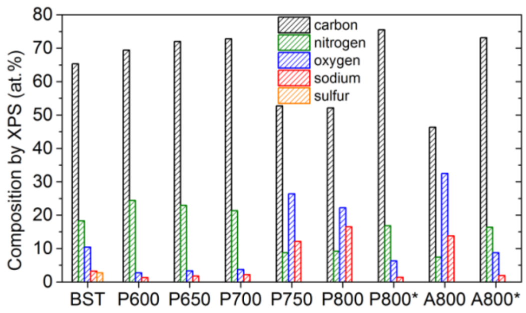

Figure S1 The elemental compositions (by XPS) of the pristine polymer foam (BST), carbon foams after pyrolysis at various temperatures (P600-P800), activation (A800) and subsequent soaking in water (P800* and A800*).

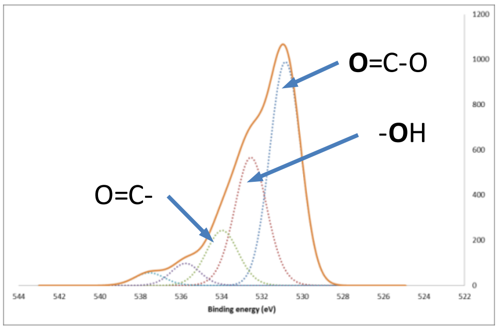

Figure S2 Resolved O1s XPS peak for A800* sample.

Table S1. Na1s X-ray photoelectron peaks of the carbon foams pyrolyzed at 750°C, 800°C and pyrolyzed and activated at 800°C (P750, P800 and A800, respectively)

**P750**		**P800**		**A800**		**Assignment**
**BE, eV**	**AC, at.%**	**BE, eV**	**AC, at.%**	**BE, eV**	**AC, at.%**	
		1069.5	2.8	1069.3	2.3	Dielectric phase
1071.5	11.7	1071.3	12.5	1071.2	11.5	Na^+
1073.6	0.4					

Catalyst particle size analysis

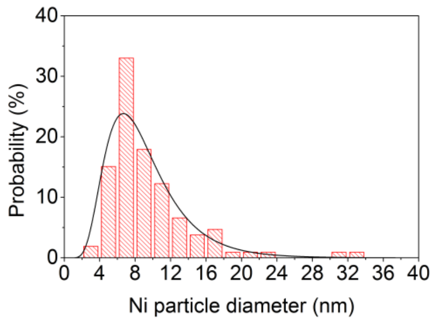

Figure S3 Ni catalyst size assessed by transmission electron microscopy (randomly counting more than 100 particles from different locations of the Ni decorated carbon foam).

Stress-strain and resistance strain curves of carbon foams

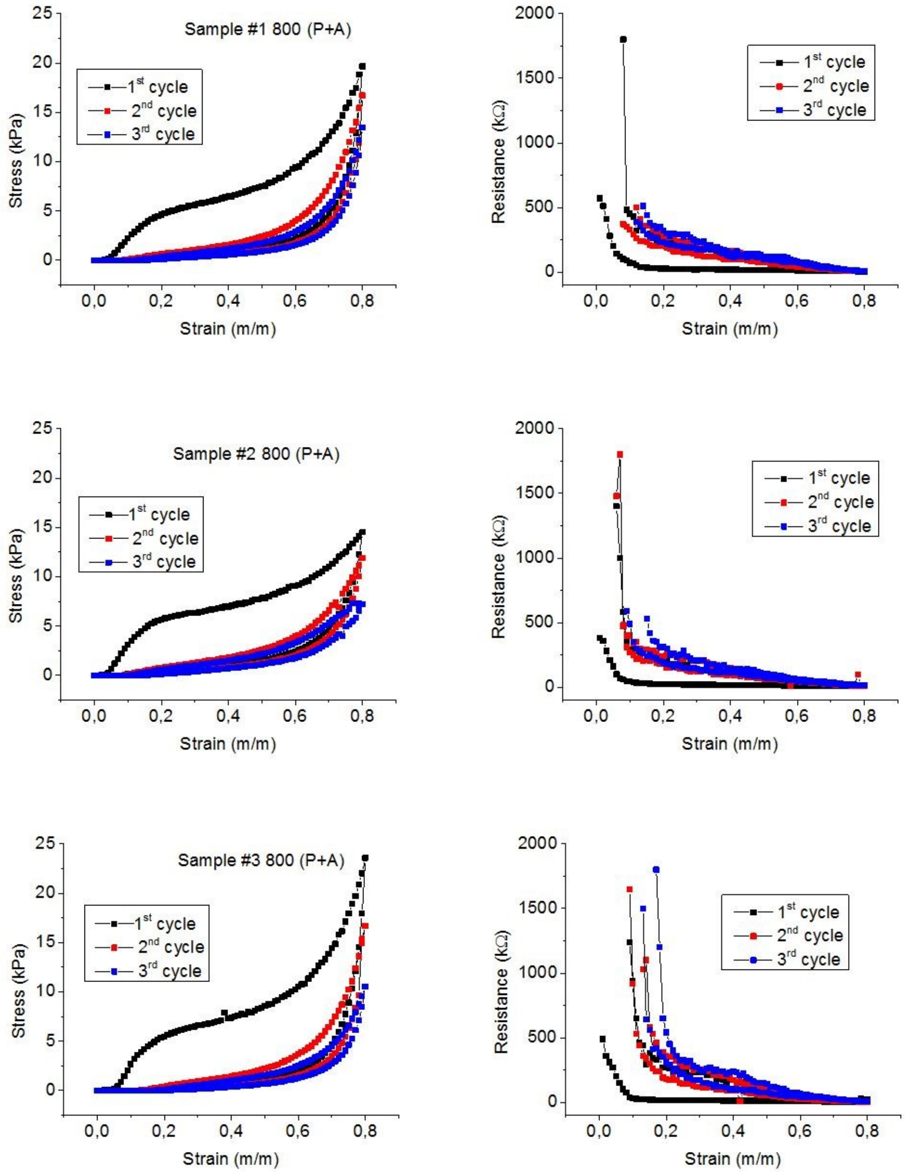

Figure S4 Stress-strain and the corresponding resistance-strain curves of carbon foams obtained by pyrolysis and subsequent activation at 800°C (size of ~10×10×10 mm^3). Each sample is subjected to three subsequent deformation cycles.

Video S1 Water droplets being repelled/absorbed by carbon foams of hydrophobic/hydrophilic nature. The wetting behavior is a function of the pyrolysis temperature.

Video S2 Crude oil stain collected and removed from the surface of water using hydrophobic carbon foam obtained by pyrolysis of melamine foam at 600°C.
